# The HUNT4 study: the validity of questionnaire-based diagnoses

**DOI:** 10.1186/s10194-019-1021-0

**Published:** 2019-06-13

**Authors:** Knut Hagen, Anders Nikolai Åsberg, Benjamin L. Uhlig, Erling Tronvik, Eiliv Brenner, Trond Sand

**Affiliations:** 1 Department of Neuromedicine and Movement science, Faculty of Medicine, Norwegian University of Science and Technology, 7489 Trondheim, Norway; 2 Norwegian Advisory Unit on Headaches, St. Olavs University Hospital, Trondheim, Norway; 3 Clinical Trial Unit, St. Olavs Hospital, Trondheim, Norway; 4 Department of Neurology and Clinical Neurophysiology, St. Olavs University Hospital, Trondheim, Norway

## Abstract

**Background:**

Questionnaire-based headache diagnoses should be validated against diagnoses made by the gold standard, which is personal interview by a headache expert. The diagnostic algorithm with the best diagnostic accuracy should be used when later analysing the data.

**Methods:**

The Nord-Trøndelag Health Study (HUNT4) was performed between 2017 and 2019. Among HUNT4 participants, a total of 232 (19.3%) out of 1201 randomly invited were interviewed by a headache expert to assess the sensitivity, specificity and kappa value of the questionnaire-based headache diagnoses.

**Results:**

The median interval between answering the headache questions and the validation interview was 60 days (95% CI 56–62 days). The best agreements were found for self-reported lifetime migraine (sensitivity of 59%, specificity of 99%, and a kappa statistic of 0.65, 95% CI 0.55–0.75), self-reported active migraine (sensitivity of 50%, specificity of 97%, and a kappa statistic of 0.55, 95% 0.39–0.71), liberal criteria of migraine (sensitivity of 64%, specificity of 93%, and a kappa statistic of 0.58, 95% CI 0.43–0.73) and ICDH3-based migraine ≥1 days/month (sensitivity of 50%, specificity of 94%, and a kappa statistic of 0.49, 95% CI 0.30–0.68). For headache suffering ≥1 days/month a sensitivity of 90%, specificity 80%, and a kappa statistic of 0.55, 95% CI 0.41–0-69 were found. For tension-type headache (TTH) ≥ 1 days/month the agreement was 0.33 (95% CI 0.17–0.49).

**Conclusion:**

The HUNT4 questionnaire is a valid tool for identifying persons with lifetime migraine, self-reported active migraine and active migraine applying liberal modified criteria. The agreement for TTH was fair.

## Introduction

A careful history taken by a face-to-face interview by a headache expert is the “gold standard” for making a valid headache diagnosis [1]. However, a self-administrated headache questionnaire is less time-consuming and costly, and commonly used in large-scale population-based studies. With questionnaire-based diagnoses, it is necessary to validate against the gold standard method [1]. Accordingly, during the last decade many validation studies have been published e.g. [2–12].

The headache part of the fourth Nord-Trøndelag Health Study (HUNT4) performed in 2017–2019 is mainly a replication of cross-sectional surveys performed in1995–1997 (HUNT2) and 2006–2008 (HUNT3) [13, 14]. Ideally, questionnaire-based headache diagnoses should be validated in a random selected sub-sample of participants during the period the survey is performed [1]. Even if a questionnaire can be shown to be valid at a particular time and in a particular area, it is not necessarily valid in other regions or at different time. Thus, in order to validate questionnaire-based headache diagnoses, a new question about lifetime migraine included, a clinical interview performed by headache experts was performed in a sub-sample of participants in HUNT4 [15]. The aim of the present study was to assess the sensitivity and specificity of questionnaire-based headache diagnoses using a personal interview as a gold standard.

## Materials and methods

### The fourth Nord-Trøndelag health survey (HUNT4)

**Fig. 1:**
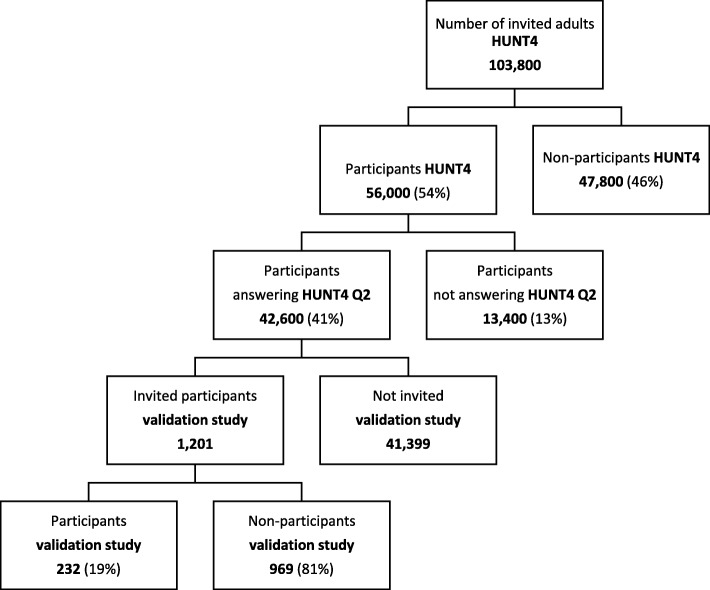
Diagram of the invited population according to type of participation based on preliminary datafrom HUNT4

All inhabitants aged 20 years or more in Nord-Trøndelag county of Norway were invited to participate in in the fourth Nord-Trøndelag Health Survey (HUNT4) in the period between August 20th 2017 and February 28th 2019. The survey included a wide number of medical topics in two extensive questionnaires (Q1 and Q2). Based on preliminary data from HUNT4, 103,800 adults received a written invitation, whereof 56,000 (54%) individuals participated and answered Q1, and 42,600 (41%) answered also Q2 (Fig. 1).

### Study population of the validation study

The present validation study is part of a subproject of HUNT4 mainly focusing on sleep disorders including an invitation to ambulatory polysomnography (PSG) and neurophysiological measurements [15]. The validation study was performed in Stjørdal community in the last week of November 2017, but some interviews were carried out in January 2018. A random sample of adults living in Stjørdal who had participated in HUNT4 and answered Q1 and Q2 received a written invitation. The invitation letter informed about an initial interview focusing on sleep and pain, and headache was not mentioned in the invitation letter [15].

### Sample size calculation

Headache diagnoses were not a part of the sample size calculation. The main goal of the sleep disorder subproject of HUNT4 was to perform at least 200 PSGs. In a previous HUNT3 PSG study, less than 17% of invited participated [16]. Thus, based on this experience, 1201 postal written invitations were sent randomly to HUNT4 participants (Fig. 1). No stratification by sex or age was performed. However, based on the HUNT3 PSG study [16], we anticipated that the participation would be higher among women than men, highest in the age group 60–69, and lowest in the age group 20–29. Based on previous headache prevalence data [13–15], a sample size of 200 was considered acceptable for the evaluation of migraine and tension-type headache (TTH) [17], but suboptimal considering headache > 15 days per month.

### Headache questionnaire in HUNT4

The Q1 included a question about lifetime migraine (Table 1). The Q2 included a total of 12 headache questions (Table 1) that were designed to determine whether the person suffered from headache and fulfilled the ICHD-3 criteria [18] for migraine or tension-type headache (TTH). The diagnoses were mutually exclusive and allowed only one diagnosis to be made in each participant. The screening question was “Have you suffered from headache during the last year?”, and only individuals who answered “yes” were asked to fill in the other headache questions. Headache sufferers were further asked to report how their headaches *usually* were regarding pain intensity, attack duration, and accompanying symptoms (Table 1). As to attack-duration the participants were not instructed to report the duration of “untreated attacks”, partly because some individuals always use attack medication for their headaches**.** In another part of the first questionnaire, the individuals were also asked to state the consumption of over-the-counter (OCT) drugs (painkillers) because of headache during the last month, with four response options: Seldom or never, 1–3 times per week, 4–6 times per week, or daily. The participants’ responses to the questionnaire in HUNT4 were unknown to the interviewers at the validation study. The main objective of the present study was to evaluate the validity of questionnaire-based information.

**Table 1:** Headache questions in the first and second questionnaire in HUNT4

Questions	Answer options
First questionnaire (Q1)	
Have you ever had migraine?	Yes or no
If yes; Age of onset	Years of age
Second questionnaire (Q2)	
Have you suffered from headache during the last year? If yes; what type of headache?	a) Yes or no Migraine or other headache
State the average number of headache days per month	Less than 1 day, 1–6 days, 7–14 days, or more than 14 days
Usually, what is the pain intensity?	Mild (does not inhibit daily activities), moderate (inhibiting, but not preventing daily activities), or severe (daily activities suspended)
For how long does the headache attack usually last?	Less than 4 h, 4 h-1 day, 1–3 days, or more than 3 days
Is the headache usually accompanied or dominated by: a) Pulsating pain? b) Pressing pain? c) One-sided pain (right or left)? d) Getting worse by physical activity? e) Nausea and/or vomiting? f) Increased sensitivity to light and sound?	a-f) Yes or no
Prior to or during headache; could you temporary have visual disturbance? (flickering lights, spots or lines, loss of vision)	Yes or no

The diagnosis of migraine in the HUNT4 questionnaire was made according to three different sets of criteria listed in Table 2. The restrictive migraine criteria set was based on ICHD-3 criteria [18], except that duration less than 4 h was accepted because it was not specifically asked for untreated headache attacks in Q2. We have previously reported that asking whether individuals had suffered from headache during the last year yielded high positive predictive value and high specificity for identifying individuals with migraine > 1 day/month [19]. Thus, because this restrictive screening question was used, the validity of migraine > 1 day/month was evaluated. For migraine with aura (MA) only visual disturbance was included in the questionnaire criteria set. Self-reported diagnosis of migraine was also considered separately because high specificity and positive predictive value of this statement were found in HUNT2 and HUNT3 [20, 21]. In accordance with the HUNT2 and HUNT3 study, self-reported migraine was integrated in the liberal migraine criteria set also used in HUNT3. The questionnaire-based diagnosis of TTH was based on the ICHD-3 criteria [18]. We have previously reported that very few subjects with infrequent TTH consider themselves as headache sufferers [19], and that a high positive predictive value and high specificity for identifying individuals with TTH > 1 day/month were found among headache sufferers [19]. Because of these findings, the validity of TTH > 1 day/month was evaluated.

**Table 2:** Three different sets of criteria for the diagnosis of migraine based on information in the questionnaire

*I. ICHD-3 modified migraine criteria (restrictive migraine criteria)*	
B. Headache attacks lasting < 72 h^a^	
C. Headache had usually at least two of the following four characteristics:	
1. Pulsating quality	
2. Unilateral location	
3. Moderate or severe pain intensity	
4. Aggravation by physical activity	
D. During headache, at least one of the following:	
1. Nausea and/or vomiting	
2. Increased sensitivity to light and sound B, C and D had to be fulfilled for the diagnosis of restrictive migraine	
*II. Migraine with aura (restrictive MA)*	
Fulfilled the restrictive migraine criteria, and reported *visual* disturbance prior to or during headache	
*III. Liberal migraine criteria (definite and probable migraine)*	
Self-reported migraine, or fulfilled the restrictive migraine criteria *IV. Lifetime migraine* Answered “yes” to the question “Have you ever had migraine?”	

^a^) Headache duration < 4 h also accepted because the participant were not asked for duration of untreated attacks in second questionnaire

To fulfil the questionnaire-based diagnosis of medication-overuse headache (MOH) the participants had to report headache > 15 days per month (i.e. chronic headache) and use of analgesics 4 times per week or more during the last month.

### Validation interview

A semi-structured interview was performed by five medical doctors (three neurologists) with special interest and competence in headache. Initially, all subjects were asked the questions “Have you ever had migraine?”. Those who answered “yes” were asked for age of onset. Later in the interview, we repeated the question regarding age of onset for each type of headache. In the interview, we focused on those who answered “yes” to the question “Have you had a headache during the last 12 months?”. We also asked an additional question identical to the screening question in Q2: “Have you suffered from headache during the last 12 months?” Individuals who reported headache during the past year were asked about frequency (average number of days per month during the last year), time span since last headache, intensity, location, aura symptoms, other migraine and cluster headache features, and use of medication. All diagnoses were based on ICHD-3 [18]. Up to three different headache types were diagnosed in those with active headache. Subjects with medication overuse headache (MOH) were also categorized according to their primary headache diagnosis.

### Ethics

This study was approved by the Regional Committee for Ethics in Medical Research (#2018/2422/Rek Midt). The participants have given written informed consent. The HUNT4 project was also approved by the Norwegian Data Inspectorate.

### Statistics

Sensitivity, specificity and Cohen’s kappa statistics with 95% CI were calculated for different headache diagnoses based on information in the questionnaires using headache experts’ headache diagnoses as a gold standard. A kappa value of ≤0.20 is considered as poor, between 0.21–0.40 as fair, between 0.41–0.60 as moderate, between 0.61–0.80 as good, and between 0.81–1.00 as very good [22].

## Results

### Participation rate in the validation interview

Among the 1201 invited participants, 239 (19.9%) agreed to participate (Fig. 1). Among these 239, seven did not attend to the interview because they were out of town, had a sick husband, were busy at work, or they had forgotten the invitation. Thus, among 1201 invited, 232 persons (19.3%) participated in the headache interview, more women (*n* = 152) than men (*n* = 80). The mean age was 58.4 years (range 22–89), and the majority (70%) of participants were in the age group 50–79 years.

### Response rate to the headache questionnaire

A total of 223 (96%) had answered the question about lifetime prevalence of migraine in Q1, whereas 224 (97%) had answered the first screening headache question in Q2.

### Time interval between response on questionnaire and interview

The mean interval between answering the Q2 and the validation interview was 60 days (95% CI 56–62 days; median 60 days, range 14–110 days).

### Validity of headache diagnoses

The sensitivity and specificity of different questionnaire-based headache diagnosis and agreement with kappa statistics between questionnaire and interview are shown in Table 3. Several diagnostic subtypes were evaluated, and the highest figures were found for lifetime migraine (kappa 0.65, 95% CI 0.55–0.75) and migraine during last year using liberal criteria (kappa 0.58, 95% CI 0.43–0.73).

**Table 3:** Sensitivity, specificity and kappa value of questionnaire-based headache criteria sets

Category	Sensitivity n (%)	95% CI	Specificity n (%)	95% CI	Kappa value	95% CI
Headache						
Headache suffering^a^	40/42 (95)	89–100	131/182 (72)	65–79	0.46	0.33–0.59
Headache suffering^a^ > 1 day/month	38/42 (90)	81–99	146/182 (80)	74–86	0.55	0.41–0.69
Headache^a^ > 15 days/month	3/8 (38)	0–81	212/216 (98)	92–100	0.38	0.15–0.61
Headache^a^ > 15 days/month with MOH^b^	1/2 (50)	0–100	222/222 (100)	99–100	0.67	0.02–1.00
Migraine						
Self-reported lifetime migraine^c^	42/71 (59)	42–72	151/152 (99)	98–100	0.65	0.55–0.75
Self-reported migraine during last year	21/42 (50)	34–66	177/182 (97)	95–99	0.55	0.39–0.71
Restrictive^d^ migraine during last year	20/42 (48)	32–63	170/182 (93)	90–96	0.45	0.28–0.62
Restrictive^d^ migraine > 1 day/month	16/32 (50)	32–68	182/192 (94)	91–97	0.49	0.30–0.68
Restrictive^d^ migraine with visual aura	7/21 (33)	11–55	191/203 (94)	91–97	0.29	0.03–0.55
Liberal^e^ migraine during last year	27/42 (64)	49–79	169/182 (93)	90–96	0.58	0.43–0.73
Tension-type headache (TTH)^f^						
TTH > 1 day/month	14/14 (100)	95–100	154/210 (73)	68–78	0.33	0.17–0.49
TTH > 15 days/month without MOH	1/6 (17)	0–60	218/218 (100)	99–100	0.28	0.00–0.90

^a^ Comparing answers of the screening question: “Have you suffered from headache during the last year” in the questionnaire and in the interview

^b^ *MOH* Medication overuse headache): Use of analgesics ≥4 times per week during the last month

^c^ Comparing questionnaire-based information about lifetime migraine with confirmed diagnoses of previous or active migraine in the interview

^d^ ICHD-3 modified migraine criteria (accepted duration less than 4 h)

^e^ Self-reported migraine, or fulfilled the restrictive criteria

^f^Tension-type headache (without co-existence of migraine)

Overall, the questionnaire-based diagnoses of active migraine using restrictive criteria (MA or MO or both) had a sensitivity of 48%, a specificity of 93%, and a kappa statistic of 0.45. Considering those with migraine > 1 days per month, the figures changed to 50%, 94%, and 0.49. Correspondingly, the sensitivity, specificity, and kappa statistics of headache suffering > 1 days per month was 90%, 80% and 0.55 (95% CI 0.41–0.69), and for TTH > 1 days per month 45%, 85%, and 0.33 (Table 3).

Few participants had headache ≥15 days/month, giving wide confidence intervals of subgroups. The sensitivity, specificity, and kappa statistics of chronic headache was 38%, 98% and 0.38 (95% CI 0.15–0.61). The corresponding kappa statistics for MOH was 0.67 (0.02–1.00) and for chronic TTH without MOH 0.28 (95% CI 0.00–0.99) (Table 3).

## Discussion

The agreement between validation interview and questionnaire-based diagnoses for lifetime migraine, self-reported active migraine and liberal criteria of migraine was good, whereas the agreement for TTH was fair [22].

### Comparison with other population-based studies

In previous studies comparing questionnaire-based and interview-based diagnosis, highly variable sensitivity and specificity for migraine and TH have been reported [12, 23]. However, because different methodological designs have been used in previous published validation studies, direct comparison should be done with caution. One important difference between studies is the wording of the initial screening question, which may vary between a neutral screening question (“Have you had a headache during the last year”), to a more restricted screening question “Have you suffered from headache during the last year” as used in the HUNT surveys [19]. Furthermore, the total number of headache questions included may vary widely. In the present HUNT4 study, a large number of other health related questions were included, allowing only 12 headache questions in the second questionnaire. In comparison, more than 65 headache questions are included in the HARDSHIP questionnaire, which is used in many recently published epidemiological studies [23].

For lifetime self-reported migraine, we found a good agreement between interview and questionnaire-based diagnoses (kappa value 0.65). Correspondingly, good agreement for lifetime migraine has previously been reported in two studies from Denmark (kappa values of 0.62 and 0.77, respectively) [24, 25].

In the validation of questionnaire-based diagnoses of active migraine different methodological strategies have been used of recruiting participants. Typically, better agreements have been reported in validation studies on migraine patients recruited from specialist practice (e.g. [5–10, 26]) than in studies recruiting participants from the general population e.g. [2–4, 20, 21, 27]. Furthermore, a better agreement may be found when the interview is performed directly after filling in the questionnaire e.g. [5, 6, 8–10] than when there is a time interval of several weeks or month e.g. [2, 4, 20, 21]. In general, with very short time interval the participants may recall their answers in the questionnaire, whereas with a long-time interval the headache condition could have changed during this period, reducing the agreement between responses in the questionnaire and clinical interview. In the guidelines it is stated that re-interviewing should be no more than 1 month after the questionnaire diagnosis [1]. In the present study, the mean time interval between questionnaire response and clinical interview was 2 months. This was not optimal, but mainly caused by an administrative delay, since the invitation letter was sent by postal mail performed by the HUNT administration instead of by telephone as done in HUNT3 [21]. The vast majority of participants were interviewed in the last week of November 2017, but 27 participants who could not attend this week had an interview approximately three weeks later. Among these, it is even more likely that the headache condition could have changed during this period. On the other hand, during the interview none of the participants reported changes in headache treatment during the last three months.

We have previously performed a similar validation study among HUNT3 participants [21]. In the present study a lower agreement between the questionnaire and clinical interview was found with respect the status headache sufferer (kappa value 0.45 versus 0.70) and chronic headache (0.38 versus 0.75) than reported in HUNT3 [21]. The better results in HUNT3 could at least partly be explained by the fact that the time span between the questionnaire and the validation study was shorter in HUNT3 (median of 45 days) [21] than in the HUNT4 study (median 60 days). In a reliability study from UK it was clearly demonstrated that the agreement for number of headache days was low after 1 month [28].

In the present study the agreement was lower for TTH than for migraine, and similar tendency has also been reported in other population-based studies [2–5, 27]. A main problem of studies based on self-administrated questionnaires is to correctly diagnose patients with the co-existence of two or more headaches, usually migraine and TTH. Some respondents with migraine may not keep the different subtypes apart when answering the questions. It may also be that many patients have somewhat atypical migraine (probable migraine), and in these patients the distinction between migraine and TTH will be difficult. Questions using the term “usually” regarding features of headache may not be ideal if one tries to make the respondent differentiate between different subtypes of headache.

Thus, the questionnaire was not optimal for estimating prevalence of TTH**.** On the other hand, high specificity (> 90) for the questionnaire-based diagnosis of migraine makes the questionnaire a valid tool for estimating migraine prevalence and to identify a population of individuals with migraine suitable for genetic studies.

### Strengths and limitations of the study

The major strength of this study was the population-based design inviting a random sample to a face-to-face interview performed by headache experts. The invitation letter did not mention that a detailed headache interview would be performed, hence a selective participation of headache patients is less likely. The previous validation study in HUNT3 was mainly performed in a different study area. Most likely, none of the participants in the present study had participated in the previous validation studies [20, 21]. Several study limitations should also be considered. The present study is a minor subproject of HUNT4 that mainly focused on sleep disorders, and participants with sleep problems were more likely to participate. Therefore, the present participants had much more insomnia than previously reported in an unselected general population (33.2% fulfilled the DSM-V diagnosis of insomnia vs. 7.9% in HUNT3 [16, 29]. The low participation rate of 19.3% could possibly partly be explained by the supplementary invitation to time-consuming ambulatory PSG and neurophysiological measurements, although the PSG-study was optional. Furthermore, most full-time workers could not attend interviews during daytime and we regrettably had no time for afternoon appointments. Finally, the low participation rate may also partly be explained by the fact that the invitation was sent by postal mail instead of an invitation directly by telephone as done in HUNT3 with an overall participation rate of 53% [21]. It should also be highlighted that 66% of the participants were women, and 70% of participants were in the age group between 50 and 79 years. The impact of the high proportion of women and elderly is difficult to interpret. However, the agreement in the present study may have been reduced because older individuals may be more likely to have change in headache condition during time compared to the younger participants.

## Conclusion

The HUNT4 questionnaire is a valid tool for diagnosing patients with lifetime migraine and active migraine applying the liberal criteria. The fair agreement for TTH makes the questionnaire-based diagnoses suboptimal for determining the prevalence of TTH in the population. However, the high specificity of the questionnaire-based diagnosis of several headache types, in particular for migraine with aura, makes the questionnaire a valid tool for diagnosing patients with migraine for genetic studies.

## Data Availability

Part of the dataset supporting the conclusions of this article is available on request to the corresponding author. Some of the data are the property of HUNT research centre and can only be accessed through direct contact with the research centre.

## Abbreviations

CI: confidence interval
HUNT: The Nord-Trøndelag Health Survey
ICHD-3: The International Classification of Headache Disorders 3rd edition
MA: Migraine with aura
MO: Migraine without aura
MOH: Medication-overuse headache
OCT: over-the-counter
PSG: Polysomnography
Q1: First questionnaire
Q2: Second questionnaire
TTH: tension-type headache

## References

[1] Stovner LJ, Al Jumah M, Birbeck GL, Gururaj G, Jensen R, Katsarava Z et al (2014) The methodology of population surveys of headache prevalence, burden and cost: principles and recommendations from the global campaign against headache. J Headache Pain 15(5) 10.1186/1129-2377-15-5 PMC3907133 24467862

[2] Rao GN, Kulkarni GB, Gururaj G, Rajesh K, Subbakrishna DK, Steiner TJ (2012). The burden of headache disorders in India: methodology and questionnaire validation for a community-based survey in KarnatakaState. J Headache Pain.

[3] Yu SY, Cao XT, Zhao G, Yang XS, Qiao XY, Fang YN (2011). The burden of headache in China: validation of diagnostic questionnaire for a population-based survey. J Headache Pain.

[4] Ayzenberg I, Katsarava Z, Mathalikov R, Chernysh M, Osipova V, Tabeeva G (2011). The burden of headache in Russia: validation of the diagnostic questionnaire in a population-based sample. Eur J Neurol.

[5] Herekar AD, Herekar AA, Ahmad A, Uqaili UL, Ahmed B, Effendi J (2013). The burden of headache disorders in Pakistan: methodology of a population-based nationwide study, and questionnaire validation. J Headache Pain.

[6] Abrignani G, Ferrante T, Castellini P, Lambru G, Beghi E, Manzoni GC (2012). Description and validation of an Italian ICHD-II-based questionnaire for use in epidemiological research. Headache.

[7] Samaan Z, Macgregor EA, Andrew D, McGuffin P, Farmer A (2010) Diagnosing migraine in research and clinical settings: the validation of the structured migraine interview (SMI). BMC Neurol 10(7) 10.1186/1471-2377-10-7 PMC2824671 20074361

[8] Shaik MM, Hassan NB, Tan HL, Bhaskar S, Gan SH (2015). Validity and reliability of the Malay version of the structured migraine interview (SMI) questionnaire. J Headache Pain.

[9] Csépány É, Tóth M, Gyüre T, Magyar M, Bozsik G, Bereczki D (2018). The validation of the Hungarian version of the ID-migraine questionnaire. J Headache Pain.

[10] van der Meer HA, Visscher CM, Engelbert RHH, Mulleners WM, Nijhuis-van der Sanden MWG, Speksnijder CM (2017). Development and psychometric validation of the headache screening questionnaire - Dutch version. Musculoskelet Sci Pract.

[11] El-Sherbiny NA, Shehata HS, Amer H, Elmazny A, Masoud M, Helmy H (2017). Development and validation of an Arabic-language headache questionnaire for population-based surveys. J Pain Res.

[12] van der Meer HA, Visscher CM, Vredeveld T, Nijhuis van der Sanden MW, Hh Engelbert R, Speksnijder CM (2019) The diagnostic accuracy of headache measurement instruments: A systematic review and meta-analysis focusing on headaches associated with musculoskeletal symptoms. Cephalalgia Apr 18 Epub ahead of print 10.1177/0333102419840777 PMC6710620 30997838

[13] Hagen K, Zwart JA, Vatten L, Stovner LJ, Bovim G (2002). Prevalence of migraine and non-migrainous headache - head-HUNT, a large population-based study. Cephalalgia.

[14] Linde M, Stovner LJ, Zwart JA, Hagen K (2011). Time trends in the prevalence of headache disorders. The Nord-Trondelag Health Studies (HUNT 2 and HUNT 3). Cephalalgia.

[15] Hagen K, Åsberg AN, Uhlig BL, Tronvik E, Brenner E, Stjern M (2018). The epidemiology of headache disorders: a face-to-face interview of participants in HUNT4. J Headache Pain.

[16] Uhlig B, Sant T, Hagen K (2019). The relationship between obstructive sleep apnea and insomnia: a population-based cross-sectional polysomnographic study. Sleep Med.

[17] Prinsen CAC, Vohra S, Rose MR, Boers M, Tugwell P, Clarke M (2016). How to select outcome measurement instruments for outcomes included in a “Core outcome set” – a practical guideline. Trials.

[18] Headache Classification Committee of the International Headache Society (IHS) The international classification of headache disorders (IHS), 3rd edition (2018). Cephalalgia 38:1–211 10.1177/0333102417738202 29368949

[19] Hagen K, Zwart JA, Aamodt AH, Nilsen KB, Bråthen G, Helde G (2008). A face-to-face interview of participants in HUNT 3: the impact of the screening question on headache prevalence. J Headache Pain.

[20] Hagen K, Zwart JA, Vatten L, Stovner LJ, Bovim G (2000). Head-HUNT: validity and reliability of a headache questionnaire in a large population-based study in Norway. Cephalalgia.

[21] Hagen K, Zwart JA, Aamodt AH, Nilsen KB, Bråthen G, Helde G (2010). The validity of questionnaire-based diagnoses: the third Nord-Trøndelag health study 2006-2008. J Headache Pain.

[22] Altman DG (1991). Inter-rater agreement. Practical statistics for medical research.

[23] Steiner TJ, Gururaj G, Andrée C, Katsarava Z, Ayzenberg I, Yu SY et al (2014) Diagnosis, prevalence estimation and burden measurement in population surveys of headache: presenting the HARDSHIP questionnaire. J Headache Pain 15(3) 10.1186/1129-2377-15-3 PMC3906903 24400999

[24] Russell MB, Rasmussen BK, Thorvaldsen P, Olesen J (1995). Prevalence and sex-ratio of the subtypes of migraine. Int J Epidemiol.

[25] Gervil M, Ulrich V, Olesen J, Russell MB (1998). Screening for migraine in the general population: validation of a simple questionnaire. Cephalalgia.

[26] Kirchmann M, Seven E, Björnsson Á, Björnssondóttir G, Gulcher JR, Stefánsson K (2006). Validation of the deCODE migraine questionnaire (DMQ3) for use in genetic studies. Eur J Neurol.

[27] Rasmussen BK, Jensen R, Olesen J (1991). Questionnaire versus clinical interview in the diagnosis of headache. Headache.

[28] Boardman HF, Thomas E, Millson DS, MacGregor EA, Laughey WF, Croft PR (2003). North Staffordshire headache survey: development, reliability and validity of a questionnaire for use in a general population survey. Cephalalgia.

[29] Uhlig BL, Engstrøm M, Ødegård SS, Hagen K, Sand T (2014). Headache and insomnia in population-based studies. Cephalalgia.

